# Livestock-associated Methicillin-Resistant *Staphylococcus aureu*s in Humans, Europe

**DOI:** 10.3201/eid1703.101036

**Published:** 2011-03

**Authors:** Brigitte A.G.L. van Cleef, Dominique L. Monnet, Andreas Voss, Karina Krziwanek, Franz Allerberger, Marc Struelens, Helena Zemlickova, Robert L. Skov, Jaana Vuopio-Varkila, Christiane Cuny, Alexander W. Friedrich, Iris Spiliopoulou, Judit Pászti, Hjordis Hardardottir, Angela Rossney, Angelo Pan, Annalisa Pantosti, Michael Borg, Hajo Grundmann, Manica Mueller-Premru, Barbro Olsson-Liljequist, Andreas Widmer, Stephan Harbarth, Alexander Schweiger, Serhat Unal, Jan A.J.W. Kluytmans

**Affiliations:** Author affiliations: National Institute for Public Health and the Environment, Bilthoven, the Netherlands (B.A.G.L. van Cleef, H. Grundmann);; VU University Medical Centre, Amsterdam, the Netherlands (B.A.G.L. van Cleef, J.A.J.W. Kluytmans);; European Centre for Disease Prevention and Control, Stockholm, Sweden (D.L. Monnet);; Radboud University Nijmegen Medical Centre, Nijmegen, the Netherlands (A. Voss);; Elisabethinen Hospital, Linz, Austria (K. Krziwanek);; Österreichische Agentur für Gesundheit und Ernährungssicherheit, Wien, Austria (F. Allerberger);; Université Libre de Bruxelles Hôpital Erasme, Brussels, Belgium (M. Struelens);; Statni Zdravotni Ustav, Praha, Czech Republic (H. Zemlickova);; Statens Serum Institut, Copenhagen, Denmark (R.L. Skov);; National Public Health Institute, Helsinki, Finland (J. Vuopio-Varkila);; Robert Koch Institute, Wernigerode, Germany (C. Cuny);; Institute of Hygiene of the University Hospital, Münster, Germany (A.W. Friedrich);; University of Patras, Patras, Greece (I. Spiliopoulou);; National Center for Epidemiology, Budapest, Hungary (J. Pászti);; Landspitali University Hospital, Reykjavik, Iceland (H. Hardardottir);; National MRSA Reference Laboratory, St. James's Hospital, Dublin, Ireland (A. Rossney);; Istituti Ospitalieri di Cremona, Cremona, Italy (A. Pan);; Istituto Superiore di Sanità, Rome, Italy (A. Pantosti);; Mater Dei Hospital, Msida, Malta (M. Borg);; Medical Faculty, Ljubljana, Slovenia (M. Mueller-Premru);; Swedish Institute for Infectious Disease Control, Solna, Sweden (B. Olsson-Liljequist);; University Hospital, Basel, Switzerland (A. Widmer);; Hôpitaux Universitaires de Genève, Geneva, Switzerland (S. Harbarth);; University Hospital Zürich, Zürich, Switzerland (A. Schweiger);; Hacettepe University, Ankara, Turkey (S. Unal);; Amphia Hospital, Breda, the Netherlands (J.A.J.W. Kluytmans)

**Keywords:** Methicillin-resistant Staphylococcus aureus, MRSA, humans, livestock, domestic animals, Europe, cross-sectional studies, bacteria, dispatch

## Abstract

To estimate the proportion of methicillin-resistant *Staphylococcus aureus* (MRSA) isolates from humans that were sequence type (ST) 398, we surveyed 24 laboratories in 17 countries in Europe in 2007. Livestock-associated MRSA ST398 accounted for only a small proportion of MRSA isolates from humans; most were from the Netherlands, Belgium, Denmark, and Austria.

Livestock-associated methicillin-resistant *Staphylococcus aureus* (MRSA) was first associated with human disease in 2003, when a MRSA clone associated with a reservoir in pigs and cattle was isolated from a human. This clone was not typable by pulsed-field gel electrophoresis with *Sma*I macrorestriction digestion and belonged to multilocus sequence type (ST) 398 (*1*). Since then, rates of MRSA ST398 carriage have been high (25%–35%) for persons in the Netherlands who have frequent contact with pigs and veal calves, but associated illness is rare (*2*). However, in Europe, Asia, and the United States, invasive infections and a hospital outbreak of MRSA ST398 have been reported (*3*). We estimated the proportion of MRSA isolates from humans in Europe in 2007 that were ST398.

## The Study

Questionnaires were mailed to 43 laboratories in 23 European countries, selected on the basis of expertise and publications about MRSA. Questions asked for level of laboratory and typing methods used, number of MRSA isolates identified in 2007, number of these isolates that were typed, and number of typed isolates that were MRSA ST398. MRSA isolates were considered to be ST398 if they 1) belonged to multilocus ST398, 2) were *spa* types t011, t034, t108, t567, t571, t588, t753, t898, t899, t1184, t1254, t1255, t1451, t1456, t1457, t2123, t2330, t2383, t2582, or t3013 (*4*; National Institute for Public Health and the Environment, unpub. data); or 3) were not typable by pulsed-field gel electrophoresis with *Sma*I macrorestriction digestion. Laboratories were asked to report data on clinical isolates only (as opposed to screening isolates) and to provide the distribution by body site.

For each laboratory, the proportion of MRSA ST398 among all typed MRSA isolates from humans and the 95% Wilson confidence interval (CI) were calculated. For laboratories that typed all MRSA isolates, χ^2^ testing compared proportions of isolates from various body sites for MRSA ST398 isolates and for other MRSA isolates.

For each country, we compared the proportions of MRSA ST398 among human MRSA isolates with number of pigs per km^2^, number of cattle <1 year of age (a surrogate for veal calves) per km^2^, and 2 indices multiplying these animal densities with human population densities. Criteria were that a laboratory had to report >100 MRSA isolates and type >25% of those isolates, thus leaving 14 national or regional laboratories from 12 countries. For Austria, data from 2 laboratories were pooled because these laboratories did not report duplicate isolates. For Denmark, only data on MRSA clinical isolates were used. Data for 2007 on midyear human population, pig production, and production of cattle <1 year of age were obtained from Eurostat (http://epp.eurostat.ec.europa.eu) except for pig production in Switzerland (Swiss Statistics, www.bfs.admin.ch) and Turkey (Turkstat, www.turkstat.gov.tr). Land area was obtained from The World Factbook (www.cia.gov/library/publications/the-world-factbook). For Germany, 1 region with high pig density was considered separately from the rest of the country. Data for this region (Eurostat regional Nomenclature of Territorial Units for Statistics code DEA3, corresponding to laboratory 8) were obtained from Eurostat, the Chamber for Agriculture Nordrhein-Westfalen: “Zahlen zur Landwirtschaft 2008” (www.landwirtschaftskammer.de/wir/pdf/zahlen-landwirtschaft-2008.pdf), and the statistical office of Nordrhein-Westfalen in Germany.

Questionnaires were received from 24 laboratories (response rate 56%) in 17 countries. Data from Malta and Slovenia and from 1 laboratory in Italy were not analyzed because these laboratories did not type MRSA isolates. Among the remaining 15 countries, 8 countries reported a combined total of 8,262 MRSA isolates with typing results, of which 142 (1.7%, 95% CI 1.5–2.0%) were MRSA ST398 (Table 1). The proportions of MRSA ST398 per country were 0–11.9%; the countries with the highest proportion were the Netherlands (11.9%), Belgium (4.7%), Denmark (1.6%), and Austria (1.4%, pooled data). The proportion of isolates from blood was significantly lower for MRSA ST398 than for other MRSA clinical isolates. No difference was observed for other body sites (Table 2).

**Table 1:** Characteristics of laboratories that reported MRSA and livestock-associated MRSA ST398 isolates from human samples, Europe, 2007*

Laboratory no.	Country	Type of laboratory	Source of MRSA isolates	No. MRSA isolates received	No. MRSA isolates typed	MRSA ST398 isolates	
						No. (%)	95% CI
1	Austria	National ref	All	523	523	0	0–0.7
2	Austria	National ref	All	586	586	16 (2.7)	1.7–4.4
3	Belgium	National ref	All	329	149	7 (4.7)	2.3–9.4
4	Czech Republic	National ref	Blood	37	10	0	0–27.8
5	Denmark	National ref	All	659	659	14 (2.1)	1.3–3.5
	Denmark	National ref	Clinical	370	370	6 (1.6)	0.7–3.5
6	Finland	National ref	All	1,323	1,323	1 (0.1)	0–0.4
7	Germany	National ref	Clinical	1,293	1,293	9 (0.7)	0.4–1.3
8	Germany	Regional ref	Clinical	866	866	37 (4.3)	3.1–5.8
9	Greece	National ref	Clinical	336	336	0	0–1.1
10	Hungary	National ref	All	365	63	0†	NA
11	Iceland	National ref	Clinical	21	21	0	0–15.5
12	Italy	National ref	Clinical	108	108	1 (0.9)	0.2–5.1
13	Ireland	National ref	Clinical	832	696	0	0–0.5
14	The Netherlands	National ref	Clinical	478	478	57 (11.9)	9.3–15.1
15	The Netherlands	Local	Clinical	12	12	3 (25.0)	8.9–53.2
16	Sweden	National ref	All	1,127	1,127	8 (0.7)	0.4–1.4
17	Switzerland	Local	Clinical	587	65	0†	NA
18	Switzerland	Regional ref	All	182	182	0	0–2.1
19	Switzerland	Regional ref	Clinical	64	64	0	0–5.7
20	Switzerland	Local	All	80	78	0	0–4.7
21	Turkey	Local	Clinical	198	60	0	0–6.0

*MRSA, methicillin-resistant *Staphylococcus aureus*; ST398, sequence type 398; CI, confidence interval; ref, reference laboratory; NA, not applicable. **†**Not reported because laboratory typed <25% of MRSA isolates.

**Table 2:** Distribution of typed MRSA ST398 and other MRSA clinical isolates, by body site, 7 European countries, 2007*

Sample source	No. (%) typed clinical isolates		p value†
	MRSA ST398, n = 113	Other MRSA, n = 3,435	
Blood	2 (1.8)	343 (10.0)	**0.004**
Respiratory tract	20 (17.7)	451 (13.1)	0.16
Skin and wound	76 (67.3)	2,312 (67.3)	0.99
Urinary tract	6 (5.3)	173 (5.0)	0.90
Other	9 (8.0)	156 (4.5)	0.09

*Only data from 9 national or regional laboratories in the 7 countries that reported clinical isolates and typed all these isolates were included. **Boldface** indicates statistical significance (p<0.05). MRSA, methicillin-resistant *Staphylococcus aureus*; ST398, sequence type 398. †χ^2^ test.

The proportion of MRSA ST398 among human MRSA isolates correlated with pig density (Spearman ρ = 0.79, p = 0.001) and with the index combining this density with human population density (Spearman ρ = 0.76, p = 0.002). The proportion of MRSA ST398 among human MRSA isolates also correlated, although less significantly, with the density of cattle <1 year of age (Spearman ρ = 0.61, p = 0.05) and with the index combining this density with human population density (Spearman ρ = 0.74, p = 0.01).

## Conclusions

Livestock-associated MRSA ST398 was reported from 8 of 15 European countries. Except for 4 countries and 1 region in Germany, the proportion of MRSA ST398 among MRSA isolates from humans was <2%, suggesting that in 2007 this livestock-associated clone contributed to only a small fraction of all MRSA in humans. A recent study of laboratories in 26 European countries during September 2006–February 2007 found no MRSA ST398 among *S. aureus* isolates from persons with invasive infections (*5*).

MRSA ST398 has been isolated from human samples from Austria (*5*), the Netherlands (*2*), Belgium (*6*), Italy (*7*), Spain (*8*), Germany (*9*), Portugal (*10*), Denmark (*11*), the Czech Republic (*12*), Sweden (*13*), and France (*14*). This study demonstrated MRSA ST398 in human samples in Switzerland and Finland. Although few data have been published on the proportion of MRSA ST398 in Europe, Springer et al. (*15*) reported that during 2006 through mid-2008, among 1,043 human MRSA isolates in Austria, 21 (2.0%) were MRSA ST398, which is similar to the proportion (1.4%, pooled data) found in our study.

Among isolates from blood, a significantly lower proportion were MRSA ST398 than other MRSA. This finding suggests that MRSA ST398 is associated with less severe disease, as indicated (*5*).

The proportion of MRSA ST398 among human MRSA isolates in European countries correlated with pig and veal calf densities and with an index combining pig or veal calf density and human population density. In addition to the well-documented risk factor of occupational exposure to pigs and veal calves, proximity of humans to pigs and veal calves may contribute to transmission of MRSA ST398 from animals to humans. However, the fact that farms are not equally distributed throughout a country may explain the higher proportion of MRSA ST398 among MRSA isolates from humans in certain European countries and regions.

Use of readily available data bears some limitations. Laboratories were not randomly selected, which could result in selection bias. However, bias was limited because most laboratories were national reference laboratories that routinely collect MRSA isolates countrywide. Also, countries may have active national or local screening policies, may select which isolates to type, and may use typing techniques that are not always fully comparable. To minimize these variations, when possible we reported on data from clinical isolates only and excluded data from laboratories that reported few isolates and did not type most MRSA isolates. We also provided a list of MRSA *spa* types that at the time of the study had been identified as corresponding to MRSA ST398. Other *spa* types and multilocus sequence types belonging to the livestock-associated MRSA clones have been recently reported (*4*) and were not included in our study.

This cross-national prevalence study found livestock-associated MRSA ST398 in human samples in several European countries. However, the relatively low proportion of MRSA ST398 among MRSA isolates from humans in most countries suggests that MRSA ST398 contributes to only a small fraction of all MRSA in humans.
